# Medicine and supplement use in infants, children, and adolescents depends on sex, age, and socioeconomic status: results of a German longitudinal population-based cohort study (LIFE Child)

**DOI:** 10.1007/s00431-022-04504-w

**Published:** 2022-05-26

**Authors:** Markus Herzig, Astrid Bertsche, Wieland Kiess, Thilo Bertsche, Martina P. Neininger

**Affiliations:** 1 Clinical Pharmacy, Institute of Pharmacy, Medical Faculty, Leipzig University and Drug Safety Center, Leipzig University and University Hospital, Leipzig, Germany; 2 University Hospital for Children and Adolescents, Center for Pediatric Research, Leipzig, Germany; 3 Neuropediatrics, University Hospital for Children and Adolescents, Rostock, Germany; 4 LIFE - Leipzig Research Center for Civilization Diseases, Leipzig University, Leipzig, Germany

**Keywords:** Cohort study, Children, Adolescents, Pharmacoepidemiology, Medicine use, Supplement use

## Abstract

Children and adolescents are exposed to medicines and supplements, but only a few studies have evaluated the actual intake in routine care. Thus, we performed a pharmacoepidemiological evaluation of a longitudinal population-based pediatric cohort study (LIFE Child) conducted at the University Hospital of Leipzig between 2014 and 2019. We analyzed all visits of the participants of the LIFE Child cohort between 1 January 2014 and 31 December 2019. Participants were asked to bring their medicines and supplements to their appointments at the study center. If they had not brought the preparations with them, attempts were made to obtain the relevant information during a telephone call after the visit to the study center. Furthermore, the participants and their parents were interviewed on medicine and supplement use and on sociodemographic and socioeconomic data during their visit to the study center. Associations of medicine and supplement use with age, sex, and socioeconomic status were analyzed using multivariate binary logistic regressions to obtain adjusted odds ratios (aOR) and 95% confidence intervals (95% *CI*). Furthermore, the number of the respective visit was included as possible confounder in the multivariate model. We included 3602 participants who visited the study center 11,906 times. The intake of 9759 medicines and supplements was recorded. Based on the evaluation of all study visits, 49% of the children and adolescents took at least one medicine or supplement. Self-medication accounted for 28% of the medicines and supplements. The prevalence of overall intake increased from 45% in 2014 to 53% in 2019 (aOR 2.63, 95% *CI* 2.23, 3.09). The prevalence was the highest (77%) in children aged 0– < 3 years, owing mainly to vitamin D. The prevalence of medicine use was higher in females (40%; aOR 1.18, 95% *CI* 1.10, 1.28) than in males (35%), owing mainly to the intake of ibuprofen and hormonal contraceptives in adolescent females. A high socioeconomic status was a predictor of lower medicine (aOR 0.80, 95% *CI* 0.68, 0.95) and higher supplement (aOR 1.47, 95% *CI* 1.09, 1.98) use.

*Conclusion*: Half of all children and adolescents took at least one medicine or supplement. The intake varied depending on age and sex. Furthermore, high socioeconomic status was associated with a decreased probability of medicine intake.

**Table:** 

**What is Known:** *• Half of all children and adolescents in Germany are exposed to medicines and supplements.* *• Data on the actual intake are scarce as most studies focus on prescribed medicines.*	
**What is New:** *• The prevalence of medicine/supplement use rose from 2014 (45%) to 2019 (53%). The prevalence was age-dependent: it was the highest in children aged < 3 years, and the lowest in children aged 6–< 9 years. Females took medicines more frequently than males.* *• High socioeconomic status was associated with lower medicine and higher supplement use. Self-medication accounted for 28% of all preparations.*

**Supplementary Information:**

The online version contains supplementary material available at 10.1007/s00431-022-04504-w.

## Introduction

To date, only a few studies have reported on the actual use of medicines and supplements in children and adolescents as most studies focus on medicine prescription data. In studies on prescription data, the prevalence of medicine use ranges from 20 to 84% depending on the study method [1–6]. However, these studies did not take into account the patient’s actual medicine intake, which may differ from the prescription, e.g., due to poor adherence, and also did not consider self-medication [1–6]. As self-medication is also associated with adverse drug reactions [7, 8], data on self-medication should also be examined to provide a complete overview of medicine and supplement use in children and adolescents. According to former studies, self-medication can range from 7 to 68%, thus constituting a large proportion of medicine use in children and adolescents [9, 10]. In addition, studies on medicine and supplement use in the pediatric population frequently investigated only specific age groups such as schoolchildren, or specific medicine groups such as antibiotics [11, 12]. Therefore, these data do not adequately reflect actual medicine and supplement use in children and adolescents, resulting in a large worldwide information gap.

In this study, we used the data of the LIFE Child cohort, a German longitudinal, prospective children’s cohort study, to obtain real-life information on the use of medicines and supplements in infants, children, and adolescents aged from 0 to < 21 years. We intended to determine whether medicine and supplement intake is associated with sociodemographic characteristics such as age, sex, or socioeconomic status (SES).

## Methods

### Study design

The data used for this investigation were collected by the “Leipzig Research Center for Civilization Diseases (LIFE) Child study” (LIFE Child cohort), an ongoing longitudinal population-based cohort study. The research center is located at the University Hospital of Leipzig (Saxony, Germany). LIFE Child aims at infants, children, and adolescents. The study is not aimed specifically at pediatric patients with diseases, but seeks to represent the population of the city of Leipzig in the cohort. More information about the study design has been published previously [13, 14]. The LIFE Child study was designed in accordance with the Declaration of Helsinki and the Ethics Committee of the University of Leipzig approved the study protocol [13]. For children under 18 years, parents were required to give written informed consent. From the age of 12 years, written informed consent was additionally obtained from the adolescents themselves.

### Recruitment

Study participants of the LIFE Child cohort were recruited using a variety of locations and communication channels, including hospitals, schools, public health centers, physicians’ offices, the internet, and other public media [13, 14]. Because participation in cohort studies depends on, for example, higher educational status and higher SES [15–17], attempts have been made to minimize bias by recruiting entire school classes, especially those from lower social backgrounds. All children and their families from the Leipzig area were welcome to participate in the LIFE Child study and public calls for participation were made regularly. For the follow-up examinations, the participants were invited to the study center each year until their 21st birthday. New participants were enrolled during the entire study period.

### Inclusion criteria

Data collection of the LIFE Child Cohort started in 2011. As a continuous, consistent data quality on medicine and supplement intake was available since 2014, and in 2020/2021 data collection was limited due to the SARS-CoV-2 pandemic; we included the data collected from 1 January 2014 to 31 December 2019 in our analyses. As LIFE Child observes adolescents up to their 21st birthday, we included all participants aged from 0 to < 21 years.

### Data collection

All data were collected at each visit for every participant. At the study center appointment, the parents (the person who accompanied the child to the examination appointment) were interviewed regarding their children’s medicine and supplement use in the 14 days prior to the appointment in the presence of the children. Older children and especially participants over 18 years, especially if they no longer live in the parents’ household, answered the questions themselves. If the parents were also present in those aged 18 years and older, the information received from the participants was completed with the parents’ answers. The participants were asked to bring their preparations to their appointment. The preparations were scanned and recorded automatically via a unique code (“Pharmazentralnummer” [18]) in an electronic database. If the participants did not bring the preparations, the information was collected using a questionnaire. Those data were manually entered into the database by a study assistant. If the participants and their parents were not able to name their medicines and supplements, efforts were made to obtain those pieces of information during a phone call after the visit to the study center. This procedure ensured that for every participant and for each visit, data on whether or not using at least one medicine or supplement in the past 14 days was available.

The data included product name, dosage form, application form, Anatomical Therapeutic Chemical classification (ATC) code, manufacturer, dosage, duration of use, and whether the medicines and supplements were recommended or prescribed by a physician. The first level of ATC code (anatomical main group) was used to group medicines and supplements.

Data collection also included the SES, which was measured at each visit using the “Winkler-Stolzenberg Index,” originally designed for the use in the German Health Interview and Examination Survey for Children and Adolescents (KiGGS) [19]. This index includes three scales comprising parental education, occupational status, and household equivalized disposable income. To calculate the total score, the highest score of both parents was used for each scale. If no value was available for one scale, the mean value of the other two scales was used according to the specifications for the use of the “Winkler-Stolzenberg Index” [20]. The resulting index ranges from 3 to 21 points and is classified into three categories: low (3–8.4 points), medium (8.5–15.4 points), and high (15.5–21.0 points). For adolescents over 18 years of age, the Winkler-Stolzenberg Index was also calculated based on parental data. Furthermore, data were collected on age, sex, monthly net household income of all household members, single parent status, number of siblings, migration background, occupational status, and educational status of the parents.

### Data processing and definitions

Since the intake of medicines and supplements can change over time, all study center visits by the participants were considered and evaluated. If two preparations with the same active ingredient were recorded for a participant at the same visit, both were included in the evaluation. This did not affect the calculation of prevalences, which were calculated as the percentages of participant visits at which the intake of at least one medicine or supplement in the past 14 days was reported.

As different specifications of the term “supplement” exist, we defined this group as follows: all preparations such as vitamins (i.e., vitamin D, vitamin C, folic acid, multivitamin preparations), essential fatty acids (i.e., omega-3 fatty acids), or minerals (i.e., calcium, magnesium, iron, zinc), regardless of the product or authorization status of the preparation in Germany, or medical/therapeutic reasons for intake. Intake of homeopathics was not included in our analysis.

Furthermore, the proportion of self-medication was examined. For this purpose, we evaluated the information on whether or not the medicines and supplements were taken on the basis of a physician’s prescription or recommendation. We defined those medicines and supplements as self-medications that were neither prescribed nor recommended by a physician.

### Statistical analysis

We used IBM SPSS Statistics 28.0.1.1 (IBM, Armonk, NY, USA) for statistical analysis and OriginPro 2019 (OriginLab, Northampton, MA, USA) for graphical visualization. Since the data for all variables considered in our analyses were newly recorded for each visit, the respective data set was evaluated for each visit. All visits to the study center were included in the analysis. Descriptive statistics were used to characterize the study population at their first visit to the study center in the study period. We report absolute and relative frequencies. We calculated median, quartiles (Q25/Q75), and min./max. for the exact age of the participants at the first visit. For further descriptive calculations, the participants were assigned to categorical age groups (0– < 3 years, 3– < 6 years, and so forth). The prevalences were calculated as the percentages of participant visits at which the intake of at least one medicine or supplement in the past 14 days was reported. This calculation included a bootstrap (replications: 2000; bias-corrected) to obtain the 95% confidence intervals (95% *CI*). Multivariate binary logistic regressions adjusted for age, sex, number of the respective visit, and SES were calculated to determine adjusted odds ratios (aOR) with 95% *CI*. For the regressions, we used the exact age of the participants. Other variables considered for regression were single parent status, number of siblings, and migration background. Variables with ≥ 40% missing values were excluded from the regressions as described in literature [21]. Variables with less than 5% missing values were included in the regressions without further adjustment procedures such as imputation [21]. If a case had a missing value in one of the included variables, it was dropped from the analysis. Thus, the regressions were only run on cases with a complete data set for the included variables. All variables were tested for multicollinearity before they were included in the multivariate regressions as multicollinear variables must not be included in the same multivariate model. According to the literature, multicollinearity was assumed at a correlation coefficient of *r* > 0.85 [22]. The data on educational status, occupational status, and monthly net equivalent household income were already reflected in the SES. Therefore, these variables were not included individually in the multivariate analysis. A *p*-value of ≤ 0.05 was considered to indicate significance.

## Results

### Characteristics of the study population

A total of 3602 participants, thereof 48% females, met the inclusion criteria (Table 1). The participants had a median age of 5.6 years at their first visit to the study center in the study period (Q25/Q75: 0.5/11.1, min./max.: 0.1/19.8). The participants visited the study center altogether 11,906 times between 1 January 2014 and 31 December 2019. The participants’ age for each year of the study period is shown in Supplementary Fig. 1. A total of 9759 medicines and supplements were recorded and included in the analyses.

**Table 1:** Sociodemographic data of participants at their first visit since 2014. The data shown were collected during the participants’ first visit to the study period. LIFE Child 2014–2019

	**Total**			**Males**				**Females**		
	***n***	**%**		***n***		**%**		***n***		**%**
**Total**	3602	100		1870		52		1732		48
**Age group in years**										
0– < 3	1372	38		726		39		646		37
3– < 6	511	14		259		14		252		15
6– < 9	469	13		247		13		222		13
9– < 12	506	14		285		15		221		13
12– < 15	474	13		241		13		233		14
15– < 18	235	6.5		106		5.7		129		7.4
18– < 21	35	1.0		6		0.3		29		1.7
**Socioeconomic status**										
Low	269	7.5		120		6.4		149		8.6
Middle	1951	54		1026		55		925		53
High	1219	34		650		35		569		33
Unknown	163	4.5		74		4.0		89		5.1
**Monthly household equivalent income**^**a**^										
≤ 787 €	429	12		206		11		223		13
788–999 €	314	8.7		164		8.8		150		8.7
1000–1190 €	414	12		225		12		189		11
1191–1400 €	413	12		221		12		192		11
1401–1667 €	620	17		311		17		309		18
≥ 1668 €	1260	35		672		36		588		34
Unknown	152	4.2		71		3.8		81		4.7
**Highest educational status of parents**										
No school certificate	18	0.5		9		0.5		9		0.5
Lower secondary education	92	2.6		41		2.2		51		2.9
Middle secondary education	674	19		328		18		346		20
Higher secondary education	1956	54		1050		56		906		52
Other	11	0.3		3		0.2		8		0.5
Unknown	851	24		439		24		412		24
**Highest occupational status of parents**										
Unemployed	131	3.6		59		3.2		72		4.2
Worker, employed, civil service	1993	55		1054		56		939		54
Self-employed	425	12		220		12		205		12
Other	160	4.4		80		4.3		80		4.6
Unknown	893	25		457		24		436		25
**Migration background**										
No	1988	55		1013		54		975		56
Yes	189	5.2		97		5.2		92		5.3
Unknown	1425	40		760		41		665		38
**Number of siblings**										
0	484	13		258		14		226		13
1	830	23		441		24		389		23
2	300	8.3		145		7.8		155		8.9
3	86	2.4		43		2.3		43		2.5
4	26	0.7		13		0.7		13		0.8
≥ 5	31	0.9		12		0.6		19		1.1
Unknown	1845	51		958		51		887		51
**Single parent status**										
No	902	25		470		25		432		25
Yes	74	2.1		42		2.2		32		1.8
Unknown	2626	73		1358		73		1268		73
**Total number of visits between 2014 and 2019**										
1	785	22		387		21		398		23
2	657	18		336		18		321		19
3	570	16		293		16		277		16
4	488	14		269		14		219		13
5	535	15		277		15		258		15
6	511	14		276		15		235		14
7	44	1.2		26		1.4		18		1.0
8	12	0.3		6		0.3		6		0.3

^a^The monthly net equivalent household income takes into account the number of family members in the household

### Variables included in the multivariate logistic regressions

For the calculation of the aOR, we identified sex, age, number of the respective visit, and SES as variables to be included in the models for the multivariate binary logistic regressions. Correlations between those variables were low (*r* < 0.30 for each combination of two variables). The variables single parent status, number of siblings, and migration background were not included in the multivariate binary logistic regressions owing to missing values ≥ 40%.

### Prevalence of medicine and supplement use

As shown in Table 2, 49% (95% *CI* 48, 50) of the infants, children, and adolescents used at least one medicine or supplement in the 14 days prior to the study appointment. The overall intake was the highest in the age group 0– < 3 years (77%, 95% *CI* 75, 78; Table 2), and the lowest in the age group 6– < 9 years (31%, 95% *CI* 29, 33). With increasing age, the prevalence reached 57% (95% *CI* 50, 63) in the age group 18– < 21 years. We observed an 8% increase in the frequency of participants reporting the intake of at least one medicine or supplement in the 14 days prior to the study appointment between 2014 and 2019 (aOR 2.63, 95% *CI* 2.23, 3.09): in 2014, the prevalence was 45% (95% *CI* 43, 47), and in 2019 it was 53% (95% *CI* 51, 55; Supplementary Table 1). The prevalences in the different age groups between 2014 and 2019 are shown in detail in Supplementary Fig. 2. Further analysis shows that 37% (95% *CI* 37, 38) of the children and adolescents took at least one medicine (Table 2). Medicine use was more frequent in females (40%, 95% *CI* 39, 41; aOR 1.18, 95% *CI* 1.10, 1.28) than in males (35%, 95% *CI* 34, 36). The use of supplements was especially high in participants aged 0– < 3 years (prevalence: 60%, 95% *CI* 59, 62; Table 2).

**Table 2:** Percentages of participant visits at which the intake of at least one medicine or supplement in the past 14 days was reported, categorized by age group. Adjusted odds ratios (aOR) were obtained using multivariate binary logistic regressions including the factors age groups, sex, number of the respective visit, and socioeconomic status. LIFE Child 2014–2019

		**Total**			**Males**		**Females**	
	***n***	**Prevalence in % (95% *CI*)**^**a**^	**aOR (95% *CI*)**^**a**^	***n***		**Prevalence in % (95% *CI*)**^**a**^	***n***	**Prevalence in % (95% *CI*)**^**a**^
**Total (medicines and supplements)**	5793	49 (48, 50)		2924		47 (45, 48)	2,869	51 (50, 52)
0– < 3	2658	77 (75, 78)	1.00 (reference)	1439		77 (75, 79)	1,219	76 (74, 78)
3– < 6	649	34 (32, 36)	0.16 (0.14, 0.18)	340		34 (31, 37)	309	34 (31, 37)
6– < 9	555	31 (29, 33)	0.14 (0.12, 0.16)	273		29 (27, 32)	282	32 (29, 35)
9– < 12	600	35 (33, 37)	0.17 (0.15, 0.19)	327		35 (32, 38)	273	35 (32, 39)
2– < 15	614	38 (36, 41)	0.19 (0.17, 0.22)	289		33 (30, 36)	325	44 (41, 47)
15– < 18	578	51 (48, 54)	0.32 (0.28, 0.38)	218		39 (35, 42)	360	63 (59, 67)
18– < 21	139	57 (50, 63)	0.34 (0.24, 0.48)	38		37 (29, 44)	101	72 (64, 78)
**Medicines**	4453	37 (37, 38)		2212		35 (34, 36)	2,241	40 (39, 41)
0– < 3	1449	42 (40, 43)	1.00 (reference)	800		43 (41, 45)	649	40 (38, 43)
3– < 6	623	33 (31, 35)	0.63 (0.56, 0.71)	324		32 (29, 35)	299	33 (30, 36)
6– < 9	522	29 (27, 31)	0.54 (0.48, 0.61)	252		27 (24, 30)	270	31 (28, 34)
9– < 12	581	34 (32, 36)	0.69 (0.61, 0.78)	320		34 (31, 37)	261	34 (31, 37)
12– < 15	584	36 (34, 39)	0.77 (0.68, 0.87)	272		31 (28, 34)	312	42 (39, 46)
15– < 18	560	49 (46, 52)	1.26 (1.09, 1.45)	209		37 (33, 41)	351	61 (57, 65)
18– < 21	134	55 (48, 61)	1.30 (0.92, 1.83)	35		34 (26, 42)	99	70 (63, 77)
**Supplements**	2334	20 (19, 20)		1215		19 (18, 20)	1,119	20 (19, 21)
0– < 3	2085	60 (59, 62)	1.00 (reference)	1101		59 (57, 61)	984	61 (59, 64)
3– < 6	51	2.7 (2.0, 3.4)	0.02 (0.02, 0.03)	29		2.9 (2.0, 3.8)	22	2.4 (1.5, 3.3)
6– < 9	47	2.6 (1.9, 3.4)	0.02 (0.01, 0.03)	27		2.9 (1.9, 3.9)	20	2.3 (1.5, 3.2)
9– < 12	35	2.0 (1.4, 2.7)	0.01 (0.01, 0.02)	15		1.6 (1.0, 2.3)	20	2.6 (1.7, 3.6)
12– < 15	50	3.1 (2.3, 4.0)	0.02 (0.02, 0.03)	24		2.8 (1.8, 3.7)	26	3.5 (2.4, 4.7)
15– < 18	52	4.6 (3.4, 5.9)	0.05 (0.03, 0.06)	15		2.7 (1.6, 3.7)	37	6.4 (4.7, 8.4)
18– < 21	14	5.7 (2.8, 8.5)	0.04 (0.02, 0.10)	4		3.8 (1.0, 7.7)	10	7.0 (4.2, 11)

^a^95% confidence interval

### Prevalence of active ingredients by sex and age

As shown in Table 3, most medicines and supplements were assigned to two ATC classes: 3211 (33%, 95% *CI* 32, 34) preparations to the “respiratory system” and 3093 (32%, 95% *CI* 31, 33) to the “alimentary tract and metabolism.” Females were more likely to take medicines from the classes “Genito-urinary system and sex hormones” (aOR 23.43, 95% *CI* 12.64, 43.42), “Systemic hormonal preparations, excluding sex hormones and insulins” (aOR 1.55, 95% *CI* 1.08, 2.23), and “Musculo-skeletal system” (aOR 1.56, 95% *CI* 1.36, 1.78). For all other ATC classes, no different intake frequencies between male and female were found. Of the recorded supplements, 96% were assigned to the class “alimentary tract and metabolism” and 3.4% to the class “blood and blood forming organs.” Table 4 shows that the five active ingredients with the highest prevalence varied between the age groups, and that 55% (95% *CI* 53, 56) of the children aged 0– < 3 years took at least one vitamin D preparation. The seasonal prevalences of antihistamines and salbutamol by month are shown in Supplementary Fig. 3.

**Table 3:** Proportion of the respective Anatomical Therapeutic Chemical classification (ATC) classes related to the total of medicines and supplements (A) and the corresponding prevalence (B). Prevalences were calculated as the percentages of participant visits at which the intake of at least one medicine or supplement of the respective ATC class was reported. Adjusted odds ratios (aOR) were obtained using multivariate binary logistic regressions including the factors age, sex, number of the respective visit, and socioeconomic status. The males were used as reference. LIFE Child 2014–2019

	**A** **Frequency**		**B** **Prevalence**			
**Name of ATC class**	***n***	**%**	**Total % (95% *CI*)**^**a**^	**Males % (95% *CI*)**^**a**^	**Females % (95% *CI*)**^**a**^	**aOR (95% *CI*)**^**a**^
Respiratory system	3211	33 (32, 34)	18 (18, 19)	18 (18, 19)	18 (17, 19)	0.99 (0.90, 1.09)
Alimentary tract and metabolism	3093	32 (31, 33)	22 (21, 23)	22 (21, 23)	22 (21,23)	1.08 (0.97, 1.20)
Musculo-skeletal system	1019	10 (9.9, 11)	8.3 (7.8, 8.9)	6.7 (6.1, 7.3)	10 (9.4, 11)	1.56 (1.36, 1.78)
Dermatologicals	813	8.3 (7.8, 8.9)	5.7 (5.3, 6.1)	5.6 (5.1, 6.2)	5.7 (5.1, 6.3)	1.02 (0.87, 1.20)
Nervous system	624	6.4 (5.9, 6.9)	4.8 (4.5, 5.2)	4.8 (4.3, 5.3)	4.9 (4.3, 5.5)	0.98 (0.82, 1.16)
Genito-urinary system and sex hormones	272	2.8 (2.5, 3.1)	2.2 (2.0, 2.5)	0.2 (0.1, 0.3)	4.5 (3.9, 5.1)	23.43 (12.64, 43.42)
Antiinfectives for systemic use	183	1.9 (1.6, 2.1)	1.5 (1.3, 1.7)	1.3 (1.0, 1.5)	1.7 (1.4, 2.0)	1.28 (0.94, 1.73)
Systemic hormonal preparations, excluding sex hormones and insulins	137	1.4 (1.2, 1.6)	1.1 (1.0, 1.3)	0.8 (0.6, 1.1)	1.5 (1.1, 1.8)	1.55 (1.08, 2.23)
Sensory organs	129	1.3 (1.1, 1.5)	1.0 (0.9, 1.2)	1.1 (0.8, 1.3)	1.0 (0.7, 1.2)	0.90 (0.62, 1.30)
Blood and blood-forming organs	116	1.2 (1.0, 1.4)	0.9 (0.7, 1.1)	0.9 (0.7, 1.1)	0.9 (0.7, 1.2)	0.91 (0.61, 1.37)
Cardiovascular system	77	0.8 (0.6, 1.0)	0.5 (0.4, 0.6)	0.5 (0.4, 0.6)	0.5 (0.3, 0.7)	0.69 (0.39, 1.22)
Antineoplastic and immunomodulating agents	36	0.4 (0.3, 0.5)	0.3 (0.2, 0.4)	0.3 (0.2, 0.4)	0.2 (0.1, 0.4)	0.78 (0.37, 1.64)
Antiparasitic products, insecticides, and repellents	31	0.3 (0.2, 0.4)	0.3 (0.2, 0.3)	0.3 (0.2, 0.4)	0.2 (0.1, 0.4)	1.15 (0.54, 2.41)
Various	13	0.1 (0.1, 0.2)	0.1 (0.1, 0.2)	0.1 (0.1, 0.2)	0.1 (0.0, 0.2)	0.68 (0.22, 2.08)
Unspecified	5	0.0 (0.0, 0.1)	-			

^a^95% confidence interval

**Table 4:** The five active ingredients with the highest prevalence rates by age group. LIFE Child 2014–2019

**Age**	**Name**	***n***	**Prevalence in % (95%** ***CI*)**^**a**^
0– < 3	Colecalciferol	1893/3471	55 (53, 56)
	Xylometazoline	414/3471	12 (11, 13)
	Acetaminophen	240/3471	6.9 (6.1, 7.7)
	Ibuprofen	231/3471	6.7 (5.9, 7.4)
	Silicones	199/3471	5.7 (5.0, 6.5)
3– < 6	Xylometazoline	157/1912	8.2 (7.0, 9.4)
	Ibuprofen	136/1912	7.1 (6.0, 8.3)
	Ivy leaves	61/1912	3.2 (2.5, 4.0)
	Combinations, thyme herb	48/1912	2.5 (1.8, 3.2)
	Combinations, herbal rhinologics for systemic use	33/1912	1.7 (1.2, 2.4)
6– < 9	Ibuprofen	105/1810	5.8 (4.7, 6.9)
	Xylometazoline	98/1810	5.4 (4.4, 6.5)
	Ivy leaves	41/1810	2.3 (1.6, 2.9)
	Salbutamol	29/1810	1.6 (1.0, 2.2)
	Salmeterol and fluticasone	29/1810	1.6 (1.0, 2.2)
9– < 12	Ibuprofen	143/1715	8.3 (7.1, 9.6)
	Xylometazoline	84/1715	4.9 (3.8, 5.9)
	Salbutamol	37/1715	2.2 (1.5, 2.9)
	Methylphenidate	30/1715	1.7 (1.2, 2.4)
	Cetirizine	29/1715	1.7 (1.2, 2.4)
12– < 15	Ibuprofen	158/1612	9.8 (8.4, 11)
	Xylometazoline	57/1612	3.5 (2.7, 4.5)
	Salbutamol	46/1612	2.9 (2.0, 3.7)
	Cetirizine	44/1612	2.7 (2.0, 3.5)
	Levothyroxine sodium	35/1612	2.2 (1.5, 2.9)
15– < 18	Ibuprofen	138/1140	12 (10, 14)
	Dienogest and ethinylestradiol	42/1140	3.7 (2.6, 4.8)
	Cetirizine	38/1140	3.3 (2.4, 4.5)
	Levothyroxine sodium	36/1140	3.2 (2.2, 4.3)
	Xylometazoline	36/1140	3.2 (2.2, 4.2)
18– < 21	Ibuprofen	37/246	15 (11, 20)
	Dienogest and ethinylestradiol	19/246	7.7 (4.5, 11)
	Chlormadinone and ethinylestradiol	13/246	5.3 (2.8, 8.1)
	Levothyroxine sodium	11/246	4.5 (2.0, 7.3)
	Levonorgestrel and ethinylestradiol	10/246	4.1 (1.6, 6.5)

^a^95% confidence interval

### Self-medication and prescription or recommendation by a physician

**Fig. 1:**
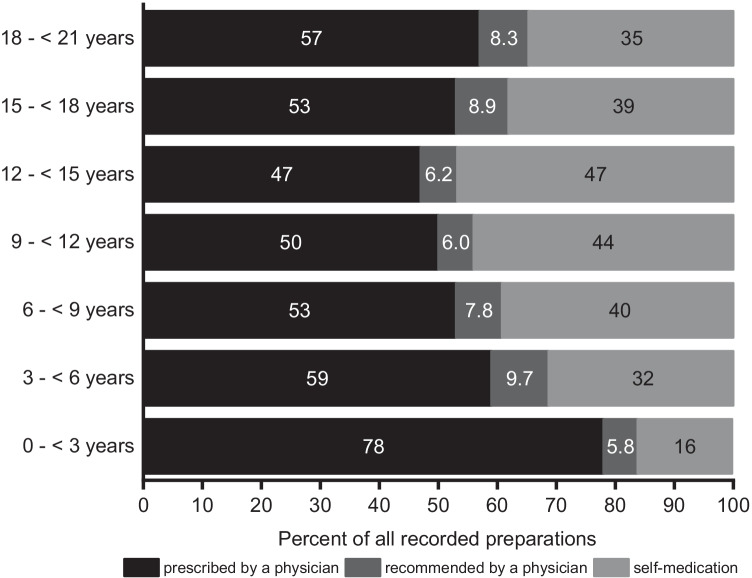
Frequency of use, depending on whether the medicines or supplements were taken on the basis of a prescription or recommendation by a physician or whether they were taken as self-medication. Information on 7485 recorded medicines and supplements was available. LIFE Child 2014–2019

For 7485 medicines and supplements (77%), information was available on whether the physician prescribed or recommended them or whether they were obtained as self-medication. Of those, 65% were prescribed by a physician. Another 6.9% were recommended by a physician and 28% were self-medication. Thirty-five percent of medicines and 8.8% of supplements were taken as self-medication. As shown in Fig. 1, self-medication accounted for 16% in participants aged 0– < 3 years, and for 47% of those aged 12– < 15 years.

### Associations between SES and intake of medicines and supplements

As shown in Table 5, a lower use of medicines was observed among participants with a high SES (aOR 0.80, 95% *CI* 0.68, 0.95) compared to participants with a low SES. The intake of supplements was more prevalent in participants with a high SES (aOR 1.47, 95% *CI* 1.09, 1.98) compared to those with a low SES. Furthermore, taking at least one self-medication was associated with high SES (aOR 1.81, 95% *CI* 1.38, 2.36) compared to low SES.

**Table 5:** Adjusted odds ratios (aOR) for the intake of at least one medicine or supplement dependent on SES, and aOR for the intake of at least one medicine or supplement based on a physician’s prescription/recommendation or on self-medication dependent on SES. aOR were obtained using multivariate binary logistic regressions including the factors age, sex, number of the respective visit, and socioeconomic status. LIFE Child 2014–2019

**Socio-economic status**	**Total (medicines and supplements)**	**Medicines**	**Supplements**	**Physician’s prescription/recommendation**	**Self-medication**
	**aOR (95% *CI*)**^**a**^	**aOR (95% *CI*)**^**a**^	**aOR (95% *CI*)**^**a**^	**aOR (95% *CI*)**^**a**^	**aOR (95% *CI*)**^**a**^
**Low**	1.00 (reference)	1.00 (reference)	1.00 (reference)	1.00 (reference)	1.00 (reference)
**Middle**	0.98 (0.84, 1.15)	0.87 (0.75, 1.02)	1.36 (1.01, 1.83)	1.03 (0.85, 1.24)	1.38 (1.06, 1.80)
**High**	0.93 (0.79, 1.10)	0.80 (0.68, 0.95)	1.47 (1.09, 1.98)	1.05 (0.87, 1.27)	1.81 (1.38, 2.36)

^a^95% confidence interval

## Discussion

### Principal findings

In this longitudinal population-based study, we analyzed real-life data to obtain a comprehensive overview of the actual use of medicines and supplements in infants, children, and adolescents. For this purpose, more than 3500 participants were included in the analysis between 2014 and 2019, providing data on almost 12,000 visits to the study center. At half of the visits, participants reported taking at least one medicine or supplement in the past 14 days. The overall intake increased by 8 percentage points between 2014 and 2019. The overall intake was age-dependent; we observed the highest prevalence rates in the age groups 0– < 3 and 18– < 21 years, and the lowest in the age group 6– < 9 years. Furthermore, medicine use was higher in females than in males. High SES was associated with a higher use of supplements and a lower use of medicines.

### Prevalence of medicine and supplement use

Only a few current studies are available worldwide that describe the use of medicines and supplements in children and adolescents. Moreover, previous studies evaluated the use of medicines and supplements mainly on the basis of prescription data. Depending on the study design and country, the prevalence rates of the prescribed medicines ranged from 20 to 84% [1–6]. Since only prescribed medicine intake was examined in those studies, they can provide only an incomplete overview, as the intake of self-medication in children and adolescents was not analyzed. More extensive data including medicines and supplements is shown, for example, in the evaluation of the Slone Survey, a random telephone survey conducted continuously in the USA between February 1998 and April 2007. In this study, 56% of children up to 12 years in the USA had taken at least one medicine or supplement in the previous 7 days [11]. For Germany, the KiGGS baseline survey (2003–2006) found a prevalence of 51% for the intake of medicines and supplements from 0 to 17 years of age (recording the past 7 days) [23]. In our study, we showed that half of the participants took at least one medicine or supplement, which is comparable to those two studies. In the follow-up survey of KiGGS (2014–2017), a lower prevalence of 36% was determined [24]. Although the methodology differed between the two KiGGS studies, the authors still found a decrease in intake based on a sensitivity analysis-corrected evaluation. It is questionable whether this is a contradiction to the increase of prevalence we observed between 2014 and 2019, since their study did not provide data for the first 3 years of life [24]. In addition, an increased outpatient utilization of pediatric health care services was observed between the KiGGS baseline survey and KiGGS follow-up survey [25]. The increase in the number of physician visits may also lead to an increased intake of medicines and supplements and, thus, may explain the increased prevalence we observed. We also investigated whether expected seasonal effects were observed in our cohort for antihistamines and salbutamol to assess the data quality of our study. For antihistamines, the expected increase in prevalence was seen in April through July, whereas the prevalence of salbutamol use remained constant throughout the year, reflecting both acute infections in winter and allergic asthma in summer.

### Age group–specific differences in prevalence rates

The prevalence rates of medicines and supplements differed between the age groups. Children aged between 0 and < 3 years were identified as the age group with the highest prevalence of supplement intake. We can clearly attribute this high prevalence to vitamin D substitution. In many countries also in Europe, vitamin D deficiency is common [26]. As a consequence, German scientific medical societies recommend vitamin D substitution for the first 1–1.5 years of life [27]. Our data positively reflects the implementation of this recommendation.

The lowest prevalence (31%) of medicine and supplement use was observed in children aged between 6 and < 9 years. From the age of 9 years, the prevalence of medicine and supplement use increased. Interestingly, between 9 and < 12 years, methylphenidate had the fourth highest prevalence. Although various studies are difficult to compare owing to different age ranges and study methods, other studies have also shown that methylphenidate is used frequently in similar age groups [28, 29].

Above the age of 14, the prevalence of medicine and supplement intake was again over 50%. Especially from this age on, we showed clear differences in prevalence rates between females and males. One of the most important contributing factors is the beginning of the use of hormonal contraceptives. Our data show that from the age group of 15 to < 18 years onwards, the combination of dienogest/ethinylestradiol, the active ingredient of a hormonal contraceptive, had the second highest prevalence. Other reports also show that hormonal contraceptive use is widespread among adolescents aged 15 years and older [30, 31]. In addition, we could show that with increasing age, females also take medicines from the “Musculo-skeletal system” class, mainly ibuprofen, more frequently than males. Studies in adults have shown that women took non-steroidal anti-inflammatory medicines (NSAIDs) more frequently than men [32, 33]. With our study, we can show that the higher consumption of analgesics in females already begins in adolescence.

### SES and medicine and supplement use

Remarkably, the probability of using a supplement increased when the SES of the participants was higher. In contrast, children and adolescents with high SES were less likely to take medicines. These findings are supported by previous studies showing that children with low SES suffer from illness more often [34–36], suggesting they also receive medicines more often than children and adolescents with a higher SES. Additionally, children and adolescents with a SES status may be oversupplied with supplements. It is questionable whether supplements are always medically necessary, especially considering that nonspecific health benefits were identified as the most important reason for use of supplements in adults and children/adolescents [37, 38].

### Strengths of the study

This study represents a current evaluation of medicine and supplement use among children and adolescents. Owing to the large study size and the implementation over a period of 6 years, we were able to evaluate a large number of participants. In contrast to other studies, not only prescription data were available but also data on the complete medicine and supplement intake, including self-medication. A special study center was set up for LIFE Child, enabling professional interviews with the children and adolescents by trained staff. This ensured a consistently high quality data. The availability of comprehensive sociodemographic data and detailed data on medicine and supplement use allowed us to provide associations between medicine and supplement use and age, sex, or SES.

### Limitations

The limited representativeness of the study cohort is a shortcoming of the LIFE Child study. LIFE Child strives to recruit as many participants as possible evenly distributed across all social milieus. Nevertheless, the cohort included more participants with middle and high SES than with low SES. This problem is common in cohort studies and limits the generalizability of study results [39–41]. Furthermore, not all participants attended all possible appointments between 2014 and 2019, leading to a potential bias. This issue is also common in longitudinal cohort studies. Possible factors for dropout and irregular visits include low education status and low household income [42, 43]. As the 0– < 3 year age group comprised the largest age group of the cohort, results may be different in cohorts with equally distributed age groups. Another limiting factor is that the parents and children may not have been able to name all medicines and supplements taken. Recall bias owing to asking about the intake during the past 14 days could occur. Furthermore, the reported medicine intake may be biased due to the various durations of treatment owing to chronic or acute illness. Last, a statistical limitation has to be considered: as the variables single parent status, number of siblings, and migration background had each ≥ 40% missing values, they could not be included in the multivariate binary logistic regression model. However, these factors could have an impact on medicine and supplement use, as different effects of these variables on health status are discussed [44–47].

## Conclusions

Our data shed light on the use of medicines and supplements in pediatric routine care. These real-life data showed that children and adolescents frequently take medicines and supplements, mainly based on a physician’s prescription or recommendation. In the longitudinal analysis, we found an increase in the overall intake in recent years. We also found that the overall intake varied depending on age. Furthermore, females took medicines more frequently than males did, mainly in adolescence because of hormonal contraceptive and ibuprofen use. We additionally found that high SES was associated with a lower medicine use and a higher supplement use. Considering the large and increasing extent of medicine and supplement use in children and adolescents, studies investigating the associated risks as well as benefits are of utmost importance, also in view of the inadequate number of clinical trials being conducted in this population group.

## Supplementary Information

Below is the link to the electronic supplementary material.

Supplementary file1 (PDF 156 KB)

## Data Availability

Not applicable.

## Abbreviations

ATC: Anatomical therapeutic chemical classification
CI: Confidence interval
KiGGS: German Health Interview and Examination Survey for Children and Adolescents
LIFE: Leipzig Research Center for Civilization Diseases
aOR: Adjusted odds ratio
SES: Socioeconomic status
