# Supplementary material for: Medicine and supplement use in infants, children, and adolescents depends on sex, age, and socioeconomic status: results of a German longitudinal population-based cohort study (LIFE Child)
Source: Eur J Pediatr. 2022 May 26;181(8):2991–3003. doi: 10.1007/s00431-022-04504-w (PMC9132604; doi:10.1007/s00431-022-04504-w)
Supplement: Supplementary file 1 — Supplementary file1 (PDF 156 KB) [file 431_2022_4504_MOESM1_ESM.pdf]

Supplementary Information

Full title: Medicine and supplement use in infants, children and adolescents depends on sex, age, and socioeconomic status: Results of a German longitudinal population-based cohort study (LIFE Child)

Journal: European Journal of Pediatrics

Authors: Markus Herzig[1], Astrid Bertsche[2,3], Wieland Kiess[2,4], Thilo Bertsche[1], Martina P Neininger[1]

[1] Clinical Pharmacy, Institute of Pharmacy, Medical Faculty, Leipzig University and Drug Safety Center, Leipzig University and University Hospital, Leipzig, Germany

[2] University Hospital for Children and Adolescents, Center for Pediatric Research, Leipzig, Germany

[3] Neuropediatrics, University Hospital for Children and Adolescents, Rostock, Germany

[4] LIFE - Leipzig Research Center for Civilization Diseases, Leipzig University, Leipzig, Germany

Corresponding author:

Thilo Bertsche, Clinical Pharmacy, Institute of Pharmacy, Medical Faculty, Leipzig University and Drug Safety Center, Leipzig University and University Hospital, Leipzig, Germany; e-mail: thilo.bertsche@uni-leipzig.de

Supplementary Figure 1: Participants' age by study year. LIFE Child 2014 - 2019.

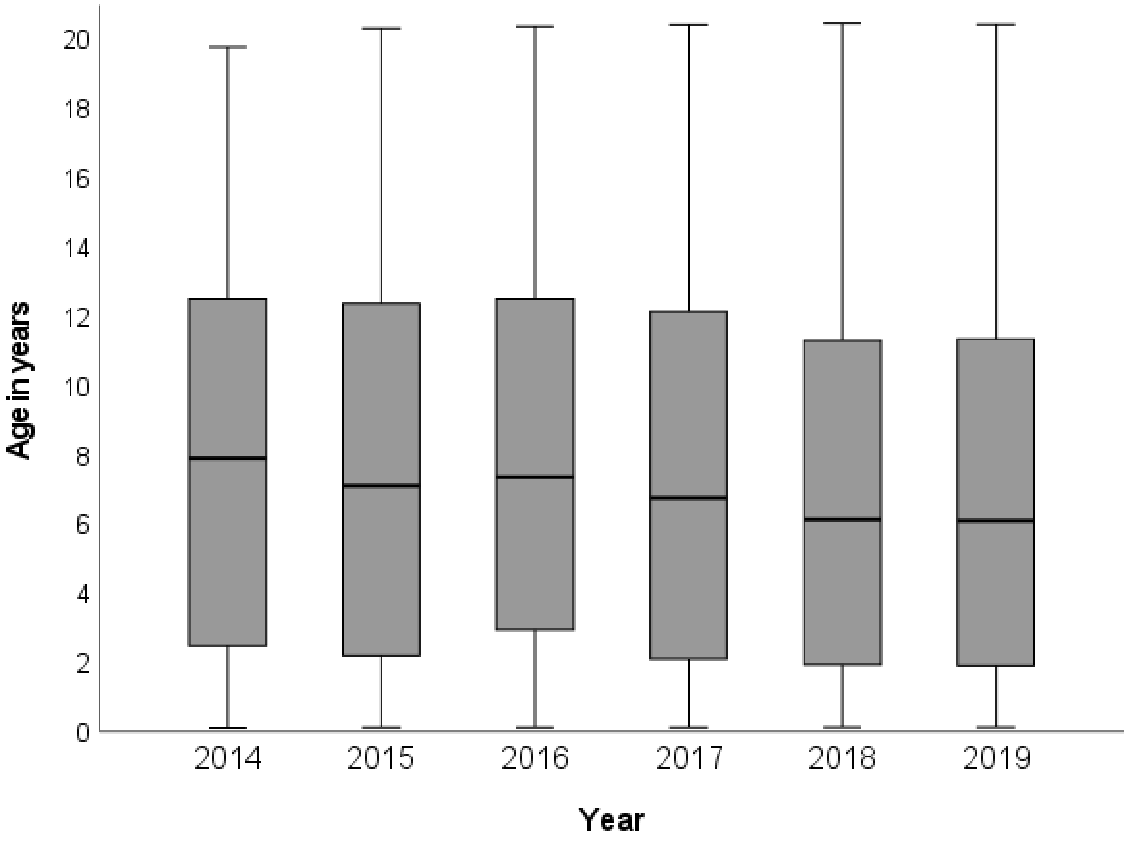

Supplementary Figure 2: Age group-related prevalence by study year. Prevalence refers to the percentages of participant visits at which the intake of at least one medicine or supplement in the past 14 days was reported. Prevalences are reported with 95% confidence intervals. LIFE Child 2014 - 2019.

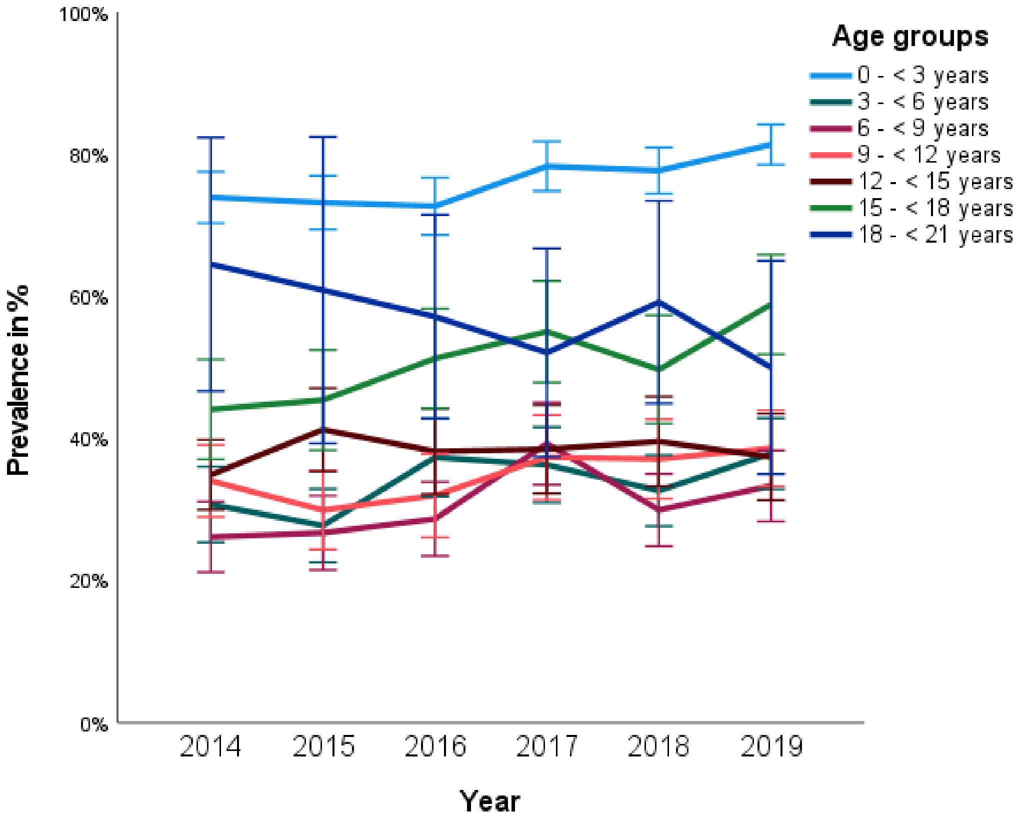

Supplementary Figure 3: Prevalences of antihistamines and salbutamol by month. Prevalence refers to the percentages of participant visits at which the intake of at least one antihistamine or salbutamol in the past 14 days was reported. Prevalences are reported with 95% confidence intervals. LIFE Child 2014 - 2019.

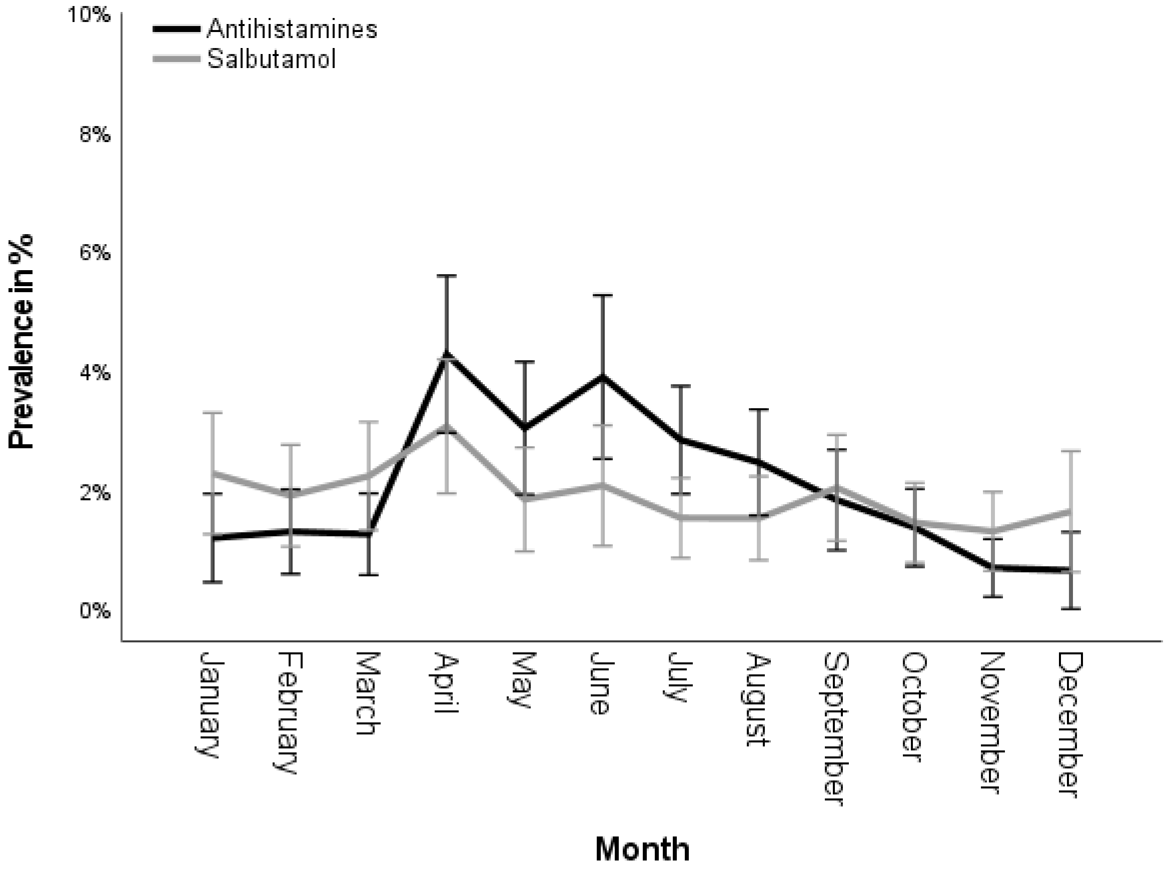

Supplementary Table 1: Results of the adjusted binary logistic regression for the use of at least one medicine or supplement by study years. Variables used for adjustment: sex, age, number of the respective visit, and socioeconomic status. LIFE Child 2014 - 2019.

**Study year**	**Prevalence in % (95% CI)[b]**	**aOR[a] (95% CI)[b]**	**p-value**
**2014**	45 (43, 47)	1.00 (Reference)	
**2015**	45 (43, 47)	1.16 (1.02, 1.33)	0.027
**2016**	47 (44, 49)	1.48 (1.28, 1.70)	< 0.001
**2017**	52 (49, 54)	2.05 (1.77, 2.38)	< 0.001
**2018**	50 (48, 52)	1.95 (1.68, 2.28)	< 0.001
**2019**	53 (51, 55)	2.63 (2.23, 3.09)	< 0.001

[a] adjusted odds ratio; [b] 95% confidence interval
